# Cross-Linked Hyaluronic Acid and Neodymium-Doped Yttrium Aluminum Garnet Laser for Oral Soft Tissue Regeneration: A Proposal of a New Surgical Technique

**DOI:** 10.7759/cureus.89045

**Published:** 2025-07-30

**Authors:** Roberta Iaria, Paolo Vescovi, Luigi Corcione, Roberto Sala, Ilaria Giovannacci

**Affiliations:** 1 Department of Medicine and Surgery, University of Parma, Parma, ITA

**Keywords:** cross-linked hyaluronic acid, nd:yag laser, oral carcinoma, oral soft tissue regeneration, porcine pericardium resorbable membrane

## Abstract

Hyaluronic acid is an endogenous molecule that acts by regulating several cellular processes, including proliferation, migration, and morphogenesis. The positive effect of this molecule on hard tissue and soft tissue regeneration is well-documented in the literature, as it actively participates in the process of tissue inflammation, thereby minimizing the damaging effects of the immune cell response on tissues. The cross-linked form of hyaluronic acid, obtained through modifications of the chemical structure of the native form, is characterized by a high capacity to resist mechanical stress and slow biodegradation, improving the clinical performance of this molecule. This article proposes a review of the literature concerning the use of cross-linked hyaluronic acid in oral soft tissue regeneration. It also reports the clinical case of an in situ carcinoma of the tongue margin managed surgically through an innovative technique that combines the use of neodymium laser surgery, cross-linked hyaluronic acid gel, and a porcine pericardium resorbable membrane. The literature was searched through the following electronic databases: MEDLINE, Scopus, Web of Science, and Cochrane Library. Follow-ups of the clinical case described showed rapid and uniform healing of the surgical site. Although the observations are limited to a single case and do not allow for objective conclusions to be drawn about the effectiveness of the proposed surgical technique, the reported results appear favorable, apparently supporting the beneficial effect of the innovative approach on the healing of surgical wounds in the oral mucosa.

## Introduction

Hyaluronic acid (also called hyaluronan), (C_14_H_21_NO_11_)_n_, is a homogeneous unbranched glycosaminoglycan (GAG) formed by a disaccharide unit (D-glucuronic acid and N-acetylglucosamine) that repeats up to 50,000 times, linked by alternating β-1,3-glycosidic and β-1,4-glycosidic bonds. Hyaluronic acid (HA) is naturally present in the body, and high amounts of this molecule are found in connective, epithelial, and neural tissues [1], where it manages processes such as cell proliferation, differentiation, migration, and adhesion by binding CD44 receptor [2].

The native form of hyaluronic acid (nHA) is characterized by a short half-life and poor resistance to mechanical forces, which justifies its limited use in clinical applications. Disadvantages associated with the clinical use of the native form of hyaluronic acid have been overcome through the development of a structurally modified form of this molecule, cross-linked hyaluronic acid (xHyA) [3].

The biological properties of the cross-linked form of hyaluronic acid justify its wide use in clinical settings. The slow degradation of xHyA promotes more prolonged action and more pronounced conditioning of the inflammatory process. These characteristics make the product potentially capable of enhancing tissue regeneration, suggesting its potential use in promoting soft tissue healing resulting from the removal of oral cavity lesions, such as the oral squamous cell carcinoma. Oral tongue squamous cell carcinoma (OTSCC) represents the most prevalent cancer of the oral cavity. According to a retrospective study conducted in 2019, OTSCC accounts for 25-40% of all oral malignancies [4].

Early stages of oral tongue squamous cell carcinoma, without lymph node involvement, are currently managed surgically through the transoral removal of the whole lesion with free margins (in the range of 0.5-1 cm in width and thickness) [5]. This study aimed to describe a step-by-step innovative approach for the surgical management of early stages of OTSCC, which involves the application of a cross-linked hyaluronic gel and the placement of a resorbable porcine pericardium membrane. Furthermore, we report results from a literature review on the clinical use of cross-linked hyaluronic acid for oral soft tissue regeneration following surgical resection of malignant lesions. Our analysis underlines a lack of literature on this specific application.

## Case presentation

**Figure 1:**
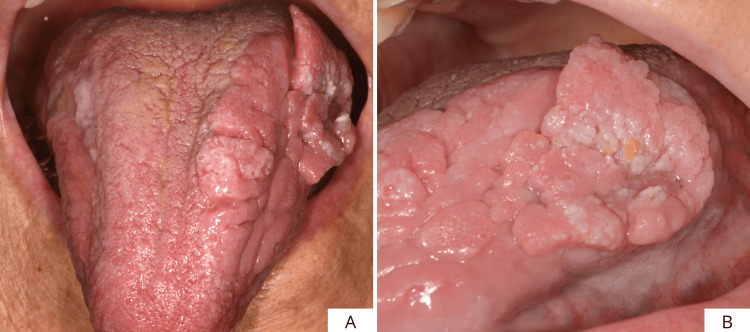
Frontal (A) and side views (B) of the lesion.

**Figure 2:**
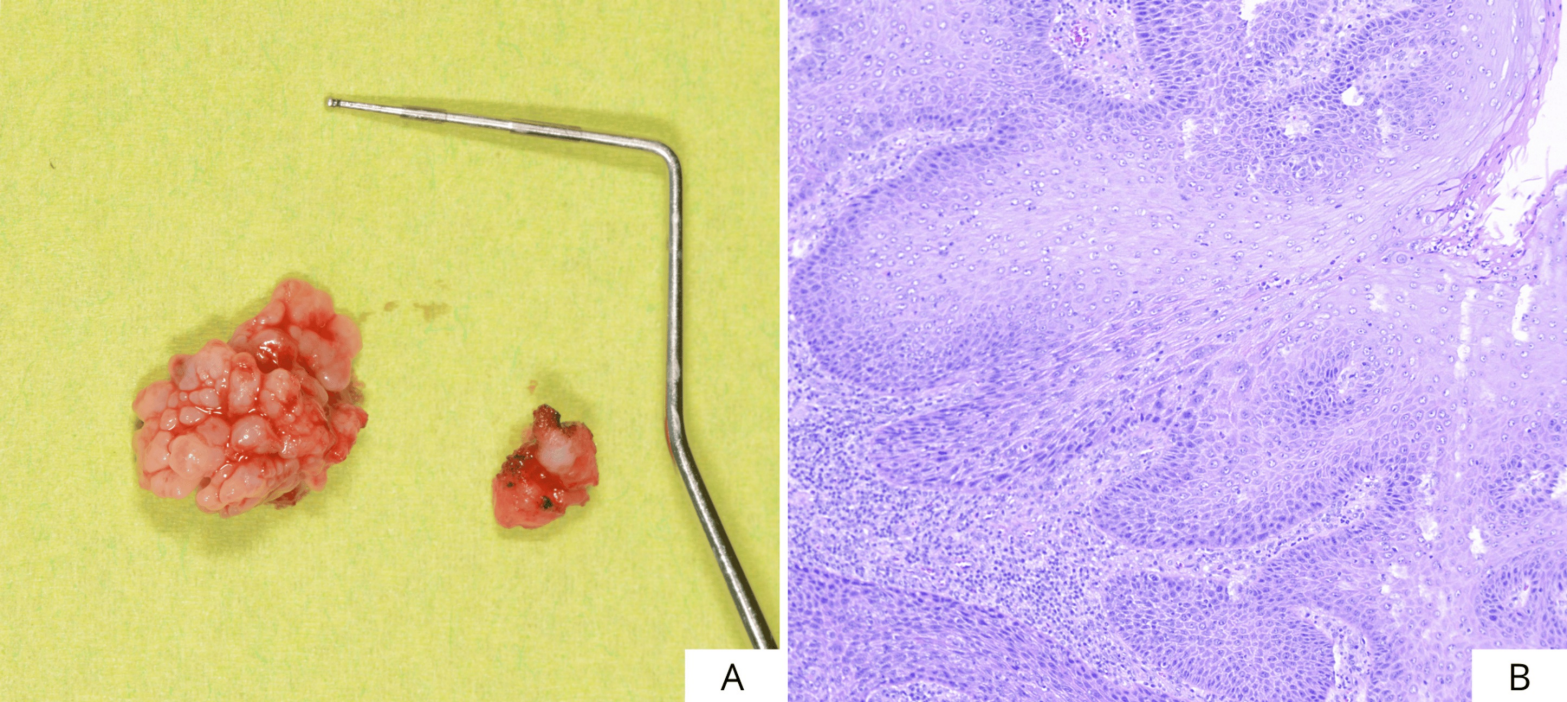
Incisional biopsy (A) and microscopic image (B) of the incisional biopsy sample: moderate-grade dysplasia.

This is a case of an 81-year-old female patient with an extensive proliferative lesion that had been present for several years, located on the left margin of the scrotal tongue, with partial involvement of the dorsum as well. The patient reported having hypertension, osteoarthritis, and rheumatoid arthritis. She also reports an allergy to the active substance of Bactrim (sulfamethoxazole and trimethoprim). No additional notable systemic conditions were reported. On clinical examination, the lesion presented an irregular, verrucous appearance, and it proved soft and non-painful on palpation. Furthermore, a small, ulcerated area was observed, probably due to trauma (Figures 1A, 1B). Because of the uncertain clinical features of the area, it was decided to perform an incisional biopsy of the lesion and a histopathological evaluation of the surgical specimen (Figures 2A, 2B).

**Figure 3:**
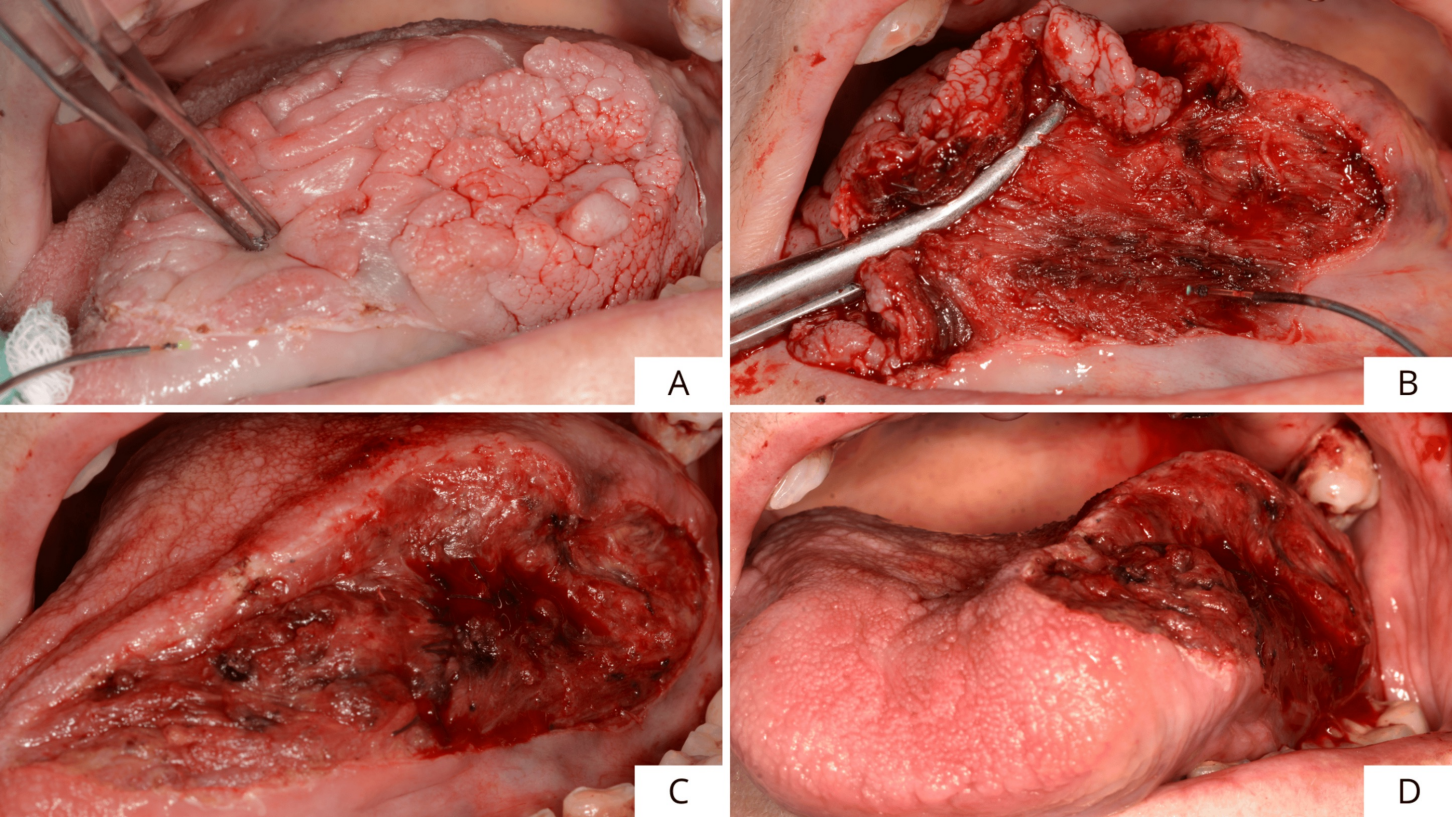
Drawing of the edges of the tissue to be removed and lesion excision conducted with Nd:YAG laser. (A) Drawing of the edges of the tissue to be removed and (B-D) lesion excision (1064 nm, output power: 3.5 W; frequency: 60 Hz; fiber diameter: 320 μm; power density 488,281 W/cm^2^).

The outcome of the incisional biopsy revealed the presence of an epitheliomatous hyperplasia with moderate-grade dysplasia, so it was decided to perform the complete surgical excision of the lesion. After loco-regional anesthesia, the margins of the tissue to be excised were identified and marked, including the lesion surrounded by safety margins (1.0 cm extension in healthy tissue) in width and depth (Figure 3A). Excision was performed using a neodymium yttrium aluminum garnet laser (Nd:YAG laser 1064 nm, output power: 3.5 W; frequency: 60 Hz; fiber diameter: 320 μm; and power density 488,281 W/cm^2^) (Figure 3B). Figures 3C, 3D show the extent of the surgical site.

**Figure 4:**
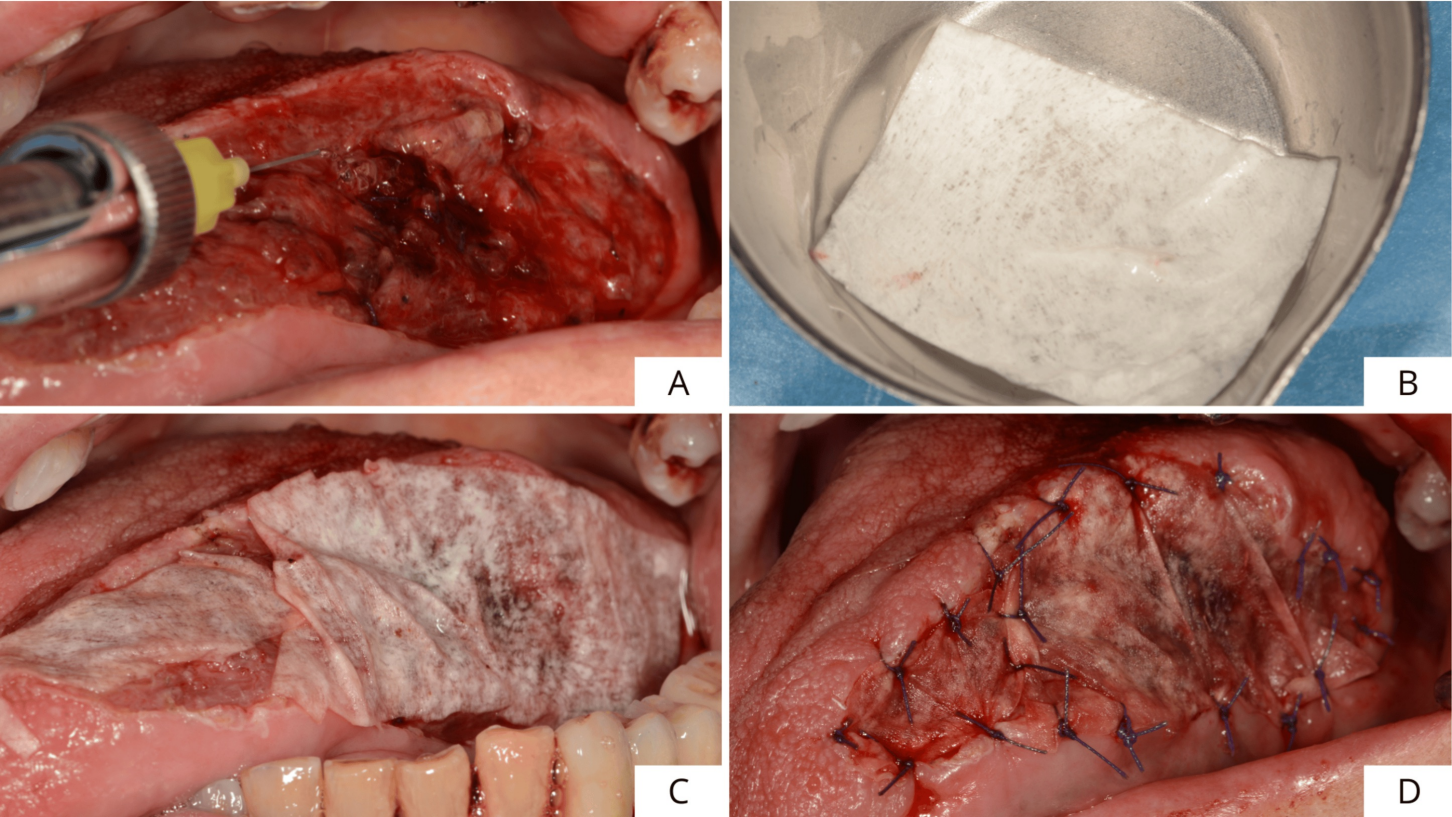
Step-by-step surgical procedure for oral soft tissue regeneration using a combination of xHyA gel and a PPRM. (A) Application of a layer of xHyA gel at the bottom of the wound, (B) membrane hydration in hyaluronic acid gel, (C) placement of PPRM to coat the surgical defect, and (D) PPRM was fixed with 5/0 Vicryl resorbable stitches. xHyA: cross-linked hyaluronic acid; PPRM: porcine pericardium resorbable membrane

Subsequently, a layer of cross-linked hyaluronic acid gel hyaDENT BG (Zurich, Switzerland: REGEDENT AG) (composition: 1.6% cross-linked hyaluronic acid, 0.2% native HA) delivered by syringe was applied (not injected) to the bottom of the wound (Figure 4A). The amount of xHyA was necessary to cover the bottom of the wound completely. Finally, a resorbable porcine pericardium membrane SMARTBRANE (Zurich, Switzerland: REGEDENT AG) (composition: native porcine pericardium), previously shaped according to the margins of the surgical defect and hydrated in saline (Figure 4B), was placed to cover the lesion (Figure 4C) and secured with 5/0 Vicryl resorbable stitches (Figure 4D).

**Figure 5:**
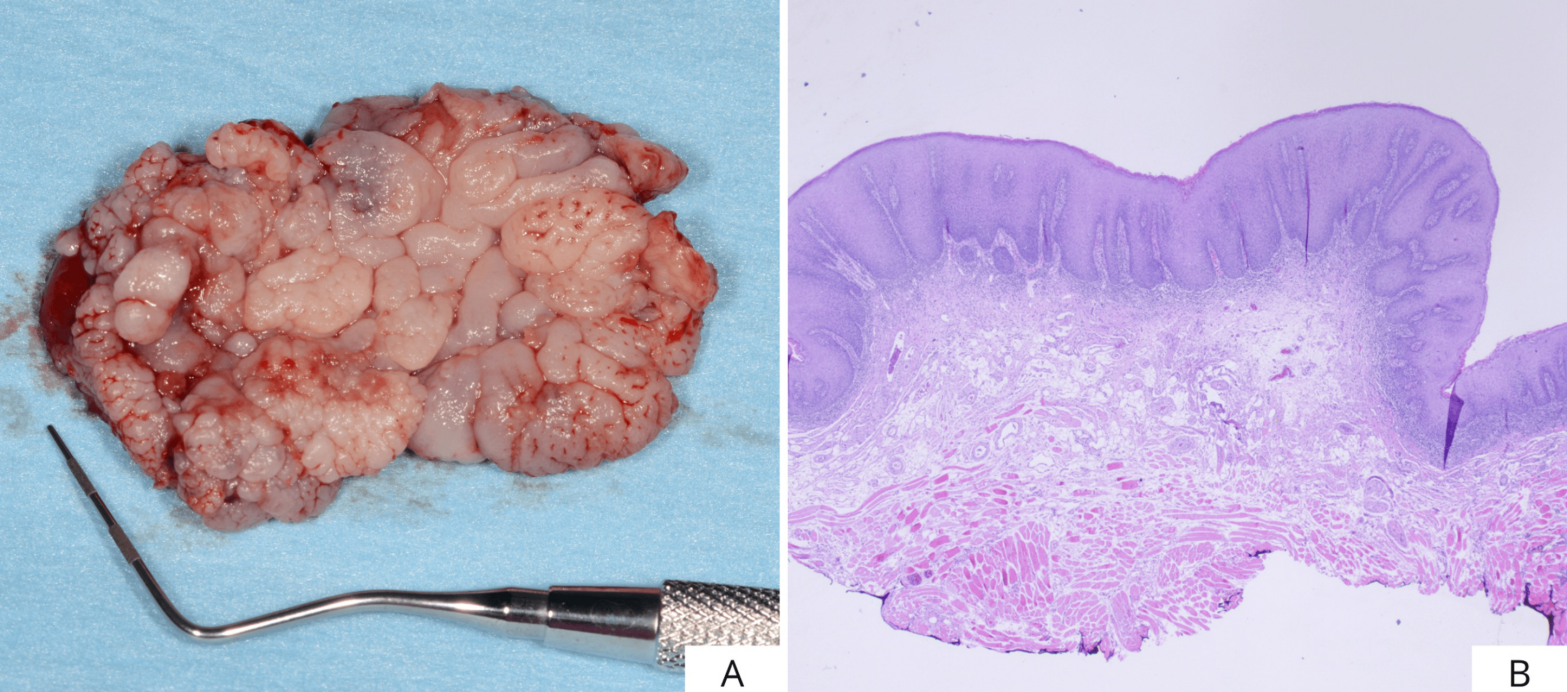
Size of removed tissue (A) and microscopic image of the surgical piece (B).

**Figure 6:**
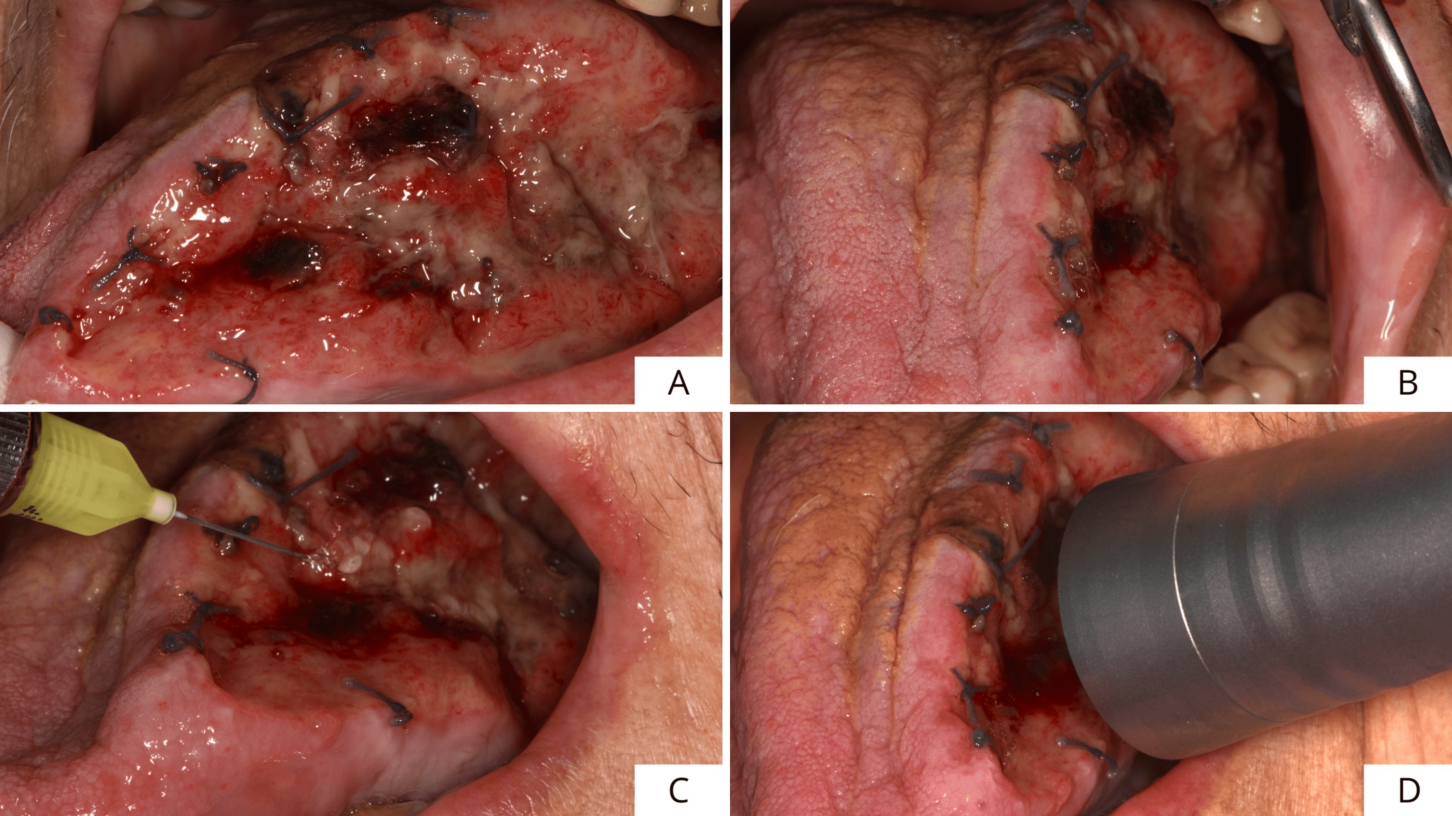
Five-day post-operative follow-up of the surgical site (A-D).

Figure 5A shows the extension of the surgical piece (4.5x3.2 cm). Histopathologic analysis of the surgical piece revealed foci of carcinoma in situ in mild/moderate dysplasia (Figure 5B). Five days after surgery, the beginning of re-epithelialization of the surgical site was observed (Figures 6A, 6B). To further promote wound healing, a second layer of cross-linked hyaluronic acid gel (Figure 6C) was applied to the surgical site. Finally, laser photobiomodulation was performed using an Nd:YAG laser (1064 nm) with the following parameters: 1.25 W power and a frequency of 15 Hz. The laser light was used in a non-focused manner with a scanning method, 2 mm from tissue, for 1 min (power density: 268.81 W/cm^2^, fluence: 14.37 J/cm^2^) and repeated five times (Figure 6D). Laser photobiomodulation therapy (LPBMT) was carried out once a week for four weeks.

**Figure 7:**
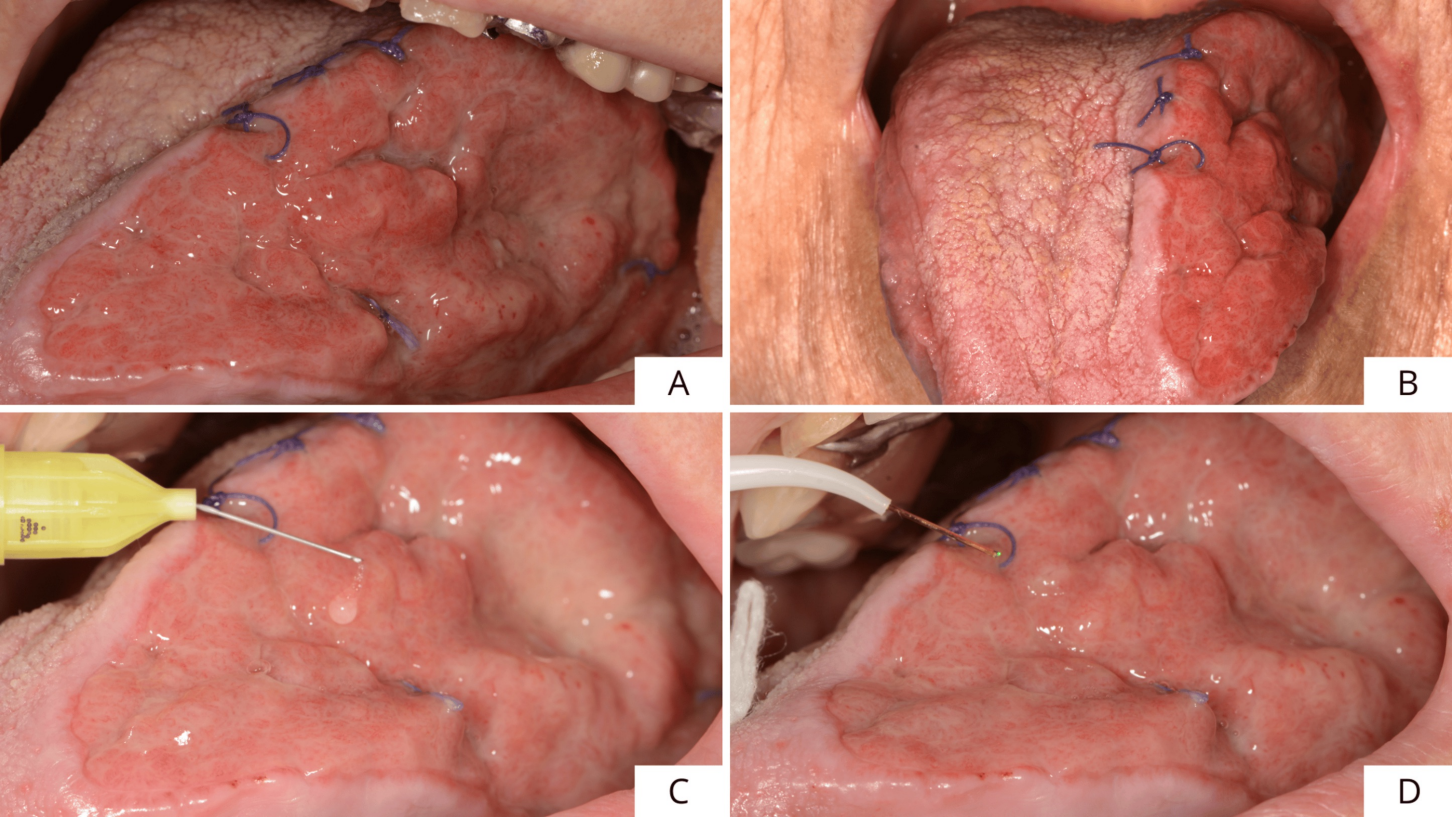
Three-weeks post-operative follow-up of the surgical site (A-D).

**Figure 8:**
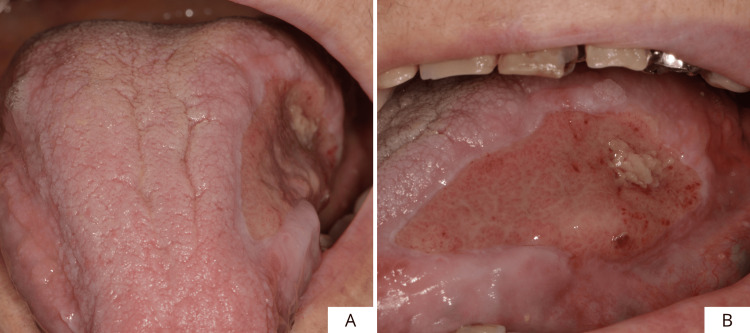
Two-month post-operative follow-up of the surgical site (A, B).

At the three-week follow-up, complete re-epithelialization of the site was finally achieved (Figures 7A, 7B). During the visit, an additional layer of cross-linked hyaluronic acid gel was applied (Figure 7C), and laser photobiomodulation was performed at the site (Figure 7D). Two months after the lesion excision, good healing of the site was observed with a reduction in lesion size in the complete absence of signs of inflammation (Figures 8A, 8B). Small areas of neoangiogenesis could be seen, confirming the usefulness of cross-linked hyaluronic acid in promoting this process (Figure 8B). Moreover, during this phase, tongue mobility has been checked, and no impairments were reported.

**Figure 9:**
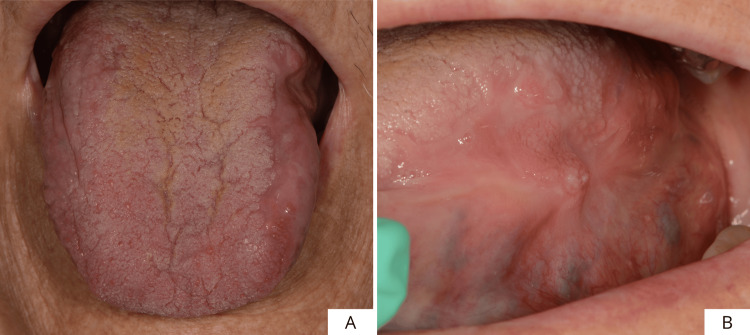
Five-month post-operative follow-up of the surgical site (A, B).

**Figure 10:**
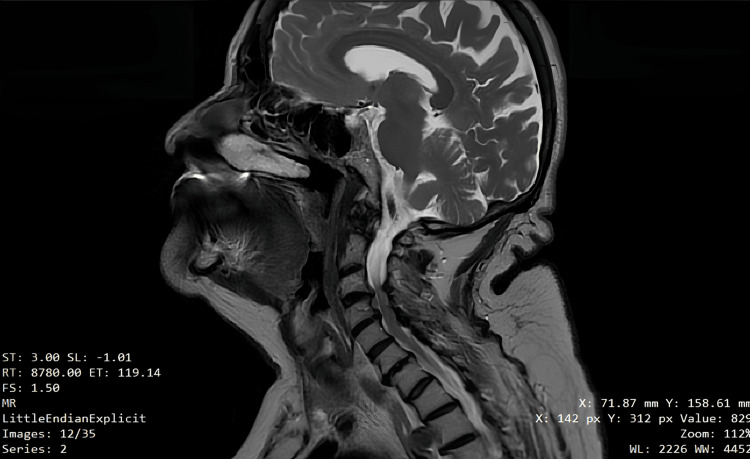
Five-month magnetic resonance imaging of the neck.

**Figure 11:**
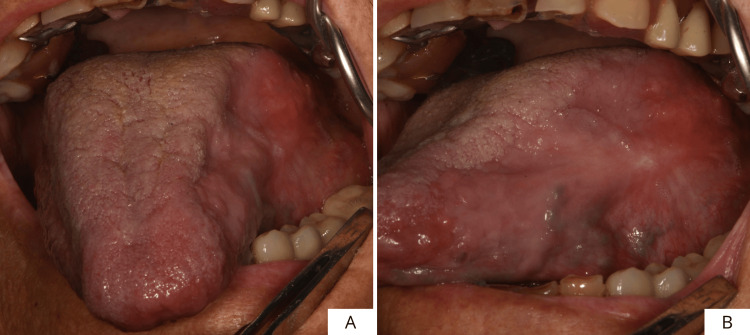
Eighteen-month post-operative follow-up of the surgical site (A, B).

At the five-month follow-up, a complete restitutio ad integrum was reported, with no loss of taste sensation (Figures 9A, 9B). Magnetic resonance imaging of the neck, performed at five months without and with contrast medium, revealed no abnormalities in the mucosal profile or lymph nodes (Figure 10). The follow-up examination performed 18 months later shows excellent healing of the surgical site (Figures 11A, 11B).

## Discussion

Literature review about the use of cross-linked hyaluronic acid for oral soft tissue regeneration

This review was conducted with the aim of answering the following main question: “Does cross-linked hyaluronic acid improve healing of post-surgical defect after resection of malignant and premalignant lesions?”

PICO statement

The PICO framework is defined as follows: population - subjects affected by malignant or potentially malignant disorders of oral mucosa that require surgical resection; intervention - application of cross-linked hyaluronic acid gel on surgical defect; comparison - conventional surgical technique without the use of cross-linked hyaluronic acid gel; and outcome - effectiveness of cross-linked hyaluronic acid in post-surgical healing.

Search strategy

**Figure 12:**
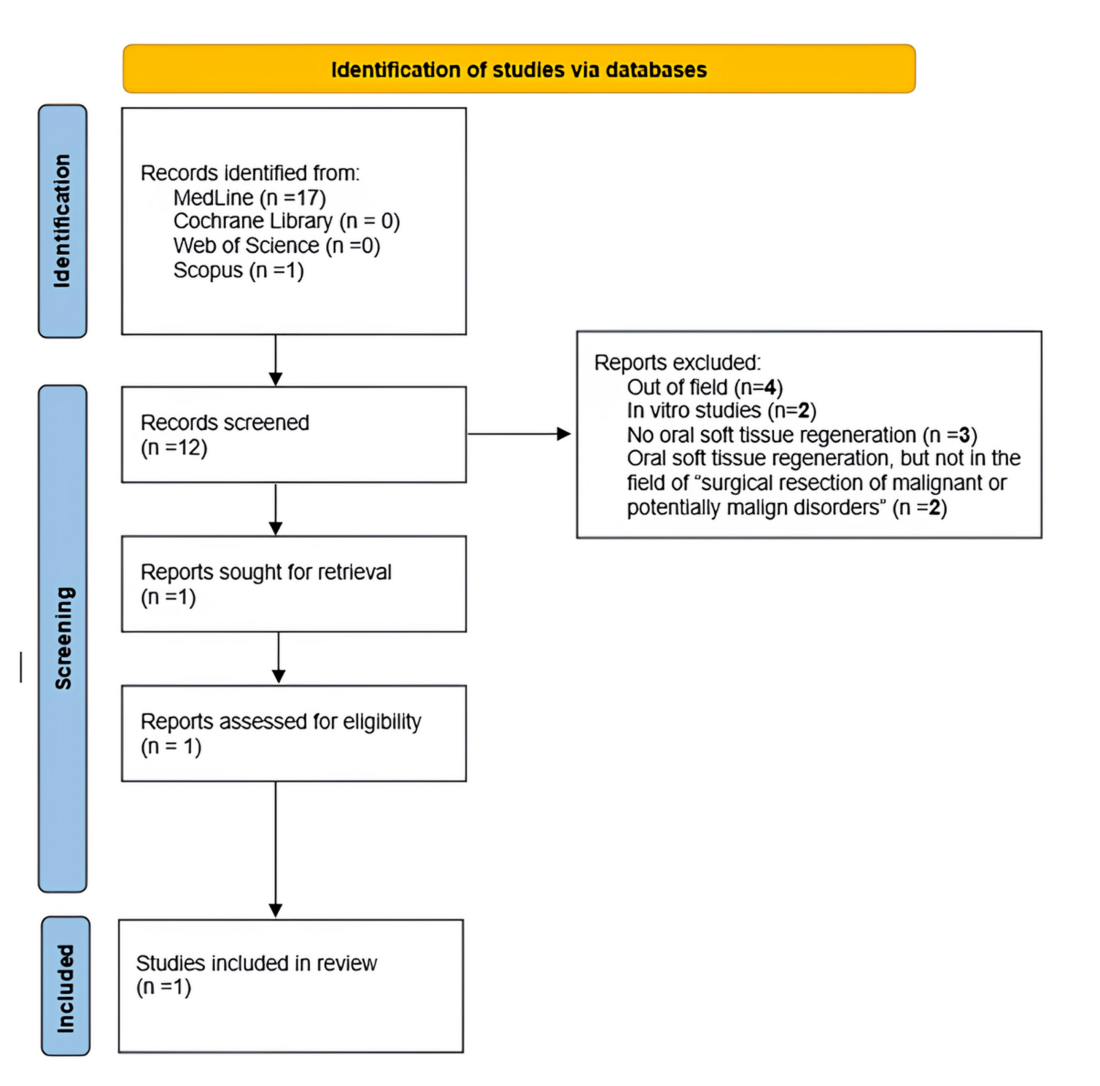
Study selection flowchart based on the 2020 PRISMA guidelines. PRISMA: Preferred Reporting Items for Systematic Reviews and Meta-Analyses

The available literature was searched in the main digital databases, including MEDLINE (1997), Web of Science (1956), Scopus (2004), and Cochrane database (1993), without time limits. In order to screen those articles that could answer the main question, the following keywords were used: “cross-linked hyaluronic acid AND oral soft tissue healing,” “cross-linked hyaluronic acid AND oral soft tissue regeneration.” Our research was conducted following the Preferred Reporting Items for Systematic Reviews and Meta-Analyses (PRISMA) statement published in 2009 (Figure 12) [6].

The selection of articles for inclusion was conducted by two reviewers and involved a rigorous screening process. Initially, articles were shortlisted based on keywords, time range, and publication type, followed by selection according to their titles. Abstracts of the screened articles were then reviewed, and studies that met the inclusion criteria were chosen. Finally, the full text of these articles was read and assessed for inclusion in the final selection (Figure 12).

Eligibility criteria

Inclusion Criteria

Papers in literature with the following characteristics were taken into consideration: studies focused on the application of cross-linked hyaluronic acid in order to promote soft tissue healing after surgical resection of malignant or potentially malignant disorders, in vivo studies, randomized clinical trials (RCT), and clinical studies, with no time limits.

Exclusion Criteria

The following were excluded: papers without an available abstract or full text, papers not in English, studies on the use of HA not in its cross-linked form, and articles on the regeneration of oral hard tissues or on the regeneration of oral soft tissues not related to the field of surgical resection of malignant or potentially malignant disorders.

Results from the literature review

Out of the 12 studies identified by searching through the aforementioned electronic databases, four studies were discarded as studies not properly related to the question of the current review [7-10]. Additionally, two studies were excluded as they were in vitro studies [11,12], and three more were discarded because they did not investigate the effect of the biomaterial on oral soft tissue regeneration [13-15]. Lastly, two studies of the screened 12 were rejected because although they evaluated the effectiveness of cross-linked hyaluronic acid in soft tissue regeneration, none of them fell within the realm of tissue healing following surgical resection of malignant or potentially malignant disorders [16,17]. The only study included is a case report, so the scientific evidence is rather weak since this type of article has a weak scientific validity [18].

What emerges from this review is that, despite the regenerative potential of the aforementioned biomaterial, there are currently no bases in the literature relating to the topic under investigation. It is clear that, in order to validate the surgical technique proposed by our research group, it is essential to conduct RCT clinical trials with large statistical populations.

Hyaluronic acid is a molecule naturally present in considerable concentrations in our body, since it represents one of the major constituents of the extracellular matrix (ECM). Specifically, significant amounts of hyaluronic acid (HA) have been found in connective, epithelial, and neural tissues [1]. It was estimated that the human skin contains over 50% of all HA in the body [19]. Hyaluronan is involved in various processes, including cell signaling, cell differentiation and proliferation, mitosis, cell mobility, morphogenesis, wound reparation, matrix organization, control of tissue hydration, and water transport and pathobiology [19,20].

HA is a hygroscopic and viscoelastic substance [21], endowed with bacteriostatic [22], anti-inflammatory [23,24], and anti-edematous actions [25]. From the available literature, its regenerative potential on tissues also emerges, mainly due to its ability to take part in and accelerate healing steps [26,27] and counteract Reactive Oxygen Species (ROS) action [28]. The quoted property of accelerating wound healing can be attributed to the effect this molecule has on the inflammatory process. The HA molecule is a very long polymer that, in certain conditions, can be segmented by the hyaluronidases, enzymes that degrade hyaluronic acid.

Currently, based on molecular weight, three different native forms of hyaluronic acid (nHA) have been identified as follows: the high-molecular-weight HA (HMW-HA, characterized by a molecular weight bigger than 5×105 Da), the medium-size HA (MMW-HA, from 2×104 to 5×105 Da), and the low-molecular-weight HA (LMW-HA, from 6×103 to 2×104 Da) [29]. The previously listed polymers differ from each other in the cellular response they are able to prompt. In particular, HMW-HA polymers exhibit immunosuppressive and anti-angiogenic actions, whereas MMW-HA is involved in ovulation, embryogenesis, regeneration, and wound repair [19].

Lastly, low-molecular-weight hyaluronic acid (LMW-HA) polymers are the ones that are able to bind to toll-like receptors (TLRs), thus promoting early inflammation and increasing the concentration of proinflammatory cytokines, such as tumor necrosis factor-α (TNF-α), interleukin-1β (IL-1β), and interleukin-8 (IL-8) [30,31]. Moreover, by activating TLR4, LMW-HA stimulates B-lymphocytes to produce interleukin-6 (IL-6) and transforming growth factor-beta (TGF-β) [32]. The action of the immune system in the early stages of the healing process is necessary in order to reach a cleansing of the wound from bacteria and damaged tissue and the induction of fibroblast production, an event that leads to the proliferation phase of the healing process [26]. Likewise, the moderation of the inflammatory process is requested to guarantee the wound reparation and to avoid tissue damage. As outlined above, hyaluronic acid in the HMW form is the one implicated in the negative control of inflammation [33]. Despite its beneficial properties, the clinical use of nHA is constrained by its high susceptibility to degradation and thus to its short in vivo half-life.

Nowadays, the cross-linked hyaluronic acid (xHyA), a new form of this GAG, has been developed by bringing structural changes in order to improve its performance and to endow it with specific properties. In particular, crosslinking of native HA polymers through either inter- or intra-functional groups of their side chains has been chemically induced using specific agents, such as divinyl sulfone (DVS) and butanediol diglycidyl ether (BDDE) [3,34]. Compared to the native form of hyaluronic acid, xHyA is characterized by a high resistance to biodegradation, lubrication, and by an increased strength to mechanical stresses, features that extend the permanence of this substance at the application site, making it ideal for clinical usage [3].

The literature shows that the clinical use of hyaluronic acid spans many different branches of medicine, rheumatology, esthetic surgery, dermatology, ophthalmology, and oral maxillofacial surgery [35]. In addition, its use is also for various tissue engineering applications. The osteogenic potential of hyaluronic acid has been widely described, and papers available in the literature suggest that this material plays an active role in bone remodeling.

An in vitro study conducted in 2023 by Tong et al. reported that HA alone promotes osteogenic differentiation in bone marrow-derived mesenchymal stem cells (BMSCs), as evidenced by an increase in osteogenic differentiation markers such as runt-related transcription factor 2 (Runx2), type I collagen (COL1), alkaline phosphatase (ALP), and osteopontin (OPN) [36]. The same study also concludes that HA inhibits the BMP-2-mediated osteogenic differentiation of BMSCs but promotes the angiogenic differentiation of human umbilical vein endothelial cells (HUVECs) [36]. In the same way, Kaneko et al., in a study published in 2015, reported that hyaluronic acid inhibits BMP-induced osteoblastic differentiation in both mouse myoblast cells (C1C12) and in mouse bone marrow cells (ST2 cells) [37].

A study conducted in 2019 with the aim of assessing the effects of low-molecular-weight hyaluronic acid on osteoblastic differentiation in human amniotic mesenchymal stem cells (hAMSCs) reported an increase in osteogenesis-associated genes RunX2, Osx, BSP, Ocn, ALP, and Col1α1 expression in cells treated with HA [38].

Due to the osteogenic potential of HA and its role in improving ligament cell viability and promoting their proliferation [11,39], application of xHyA in dentistry was widely described and documented in the periodontal field for non-surgical periodontal therapy [25], in guided bone regeneration GBR [40,41], to manage gingival recessions [42,43], to treat peri-implantitis [44], and in sinus floor augmentation [45]. Our research conducted across four databases indicates that, to date, the use of cross-linked hyaluronic acid in promoting oral soft tissue regeneration has been reported in only one article.

## Conclusions

Despite its limitations, the anti-inflammatory potential and proliferative effect on keratinocytes and fibroblasts assessed in in vitro studies suggest the possibility of using cross-linked hyaluronic acid to improve the healing of oral soft tissues. In the present case, the application of cross-linked hyaluronic acid gel was associated with favorable healing outcomes, including rapid tissue repair, minimal post-operative discomfort, absence of inflammation at follow-up, preservation of function, and good patient tolerability. Notwithstanding the encouraging results reported in this article, further studies are necessary in order to confirm the usefulness of this method in promoting soft tissue regeneration.
